# Nanomedicine-Enhanced Radiotherapy for Glioblastoma: Advances in Targeted Therapy and Adaptive Treatment Strategies

**DOI:** 10.3390/pharmaceutics17040508

**Published:** 2025-04-11

**Authors:** Kamila Rawojć, Mansoor M. Ahmed, Ayesha Mukhtiar, Magdalena Łukowiak, Kamil Kisielewicz

**Affiliations:** 1 National Institute of Oncology, Maria Sklodowska-Curie Memorial Institute, 31-115 Cracow, Poland; 2 Albert Einstein College of Medicine, Montefiore Einstein, New York, NY 10461, USA; 3 Seneca Valley High School, Germantown, MD 20874, USA; 4 Department of Medical Physics, Pomeranian Medical University, 70-204 Szczecin, Poland

**Keywords:** glioblastoma multiforme (GBM), nanomedicine, tumor microenvironment (TME), golden nanopeanuts, immunotherapy, proton therapy, stimuli-responsive nanoparticles, radiosensitization, blood–brain barrier (BBB), adaptive radiotherapy, immunogenic cell death (ICD), personalized oncology, redox-responsive nanocarriers, hypoxia-targeting nanomedicine

## Abstract

Glioblastoma multiforme remains one of the most aggressive and treatment-resistant brain tumors that necessitate innovative therapeutic approaches. Nanomedicine has emerged as a promising strategy to enhance radiation therapy by improving drug delivery, radiosensitization, and real-time treatment monitoring. Stimuli-responsive nanoparticles can overcome limitations of the blood–brain barrier, modulate tumor microenvironment, and facilitate targeted therapeutic interventions. The integration of nanotechnology with proton and X-ray radiotherapy offers improved dose precision, enhanced radiosensitization, and adaptive treatment strategies. Furthermore, Artificial Intelligence-driven nanoparticle designs are optimizing therapeutic outcomes by tailoring formulations to tumor-specific characteristics. While promising, clinical translation remains a challenge that requires rigorous validation to ensure safety and efficacy. This review highlights advancements in nanomedicine-enhanced radiotherapy and future directions for glioblastoma multiforme treatment.

## 1. Introduction

Glioblastoma multiforme (GBM), classified as a grade IV astrocytoma by the World Health Organization (WHO), remains one of the deadliest and most treatment-resistant primary brain tumors. Despite decades of intensive research, GBM continues to evade durable control due to its diffuse infiltration, rapid proliferation, and profound molecular and cellular heterogeneity. Current standard-of-care therapy, which includes maximal surgical resection, radiation therapy (RT), and concurrent temozolomide (TMZ), provides only modest survival benefits, with a median overall survival (OS) of 15–16 months and a five-year survival rate of less than 10%. This grim prognosis highlights the urgent need for innovative therapeutic strategies that can overcome GBM’s inherent resistance mechanisms.

A significant shift in GBM research focuses on the tumor microenvironment (TME) as a critical determinant of therapeutic response. The GBM TME is characterized by a highly immunosuppressive landscape, hypoxia, acidic pH, elevated glutathione (GSH) levels, and dysregulated metabolic signaling, all of which contribute to resistance against traditional therapies. These microenvironmental features present unique opportunities for targeted interventions, particularly through the development of stimuli-responsive nanomedicines designed to exploit these pathological hallmarks for selective and controlled drug delivery. Additionally, the interaction between GBM and its surrounding stromal, vascular, and immune components necessitates an integrative therapeutic approach that addresses tumor-host crosstalk.

### 1.1. Redefining Nanomedicine for GBM: Hypothesis and Approach

We propose that stimuli-responsive nanocarriers, designed to interact dynamically with the TME, represent a transformative approach to GBM therapy. Unlike traditional nanotherapeutics that rely on passive targeting via the enhanced permeability and retention (EPR) effect, stimuli-responsive systems activate precisely in response to tumor-specific cues such as hypoxia, low pH, or high GSH concentrations. These activatable platforms not only deliver therapeutic agents but also reprogram the TME to enhance radiosensitivity and reverse immunosuppressive signaling. For example, nanoparticles engineered to release immunomodulators under hypoxic conditions could amplify radiation-induced tumor cell death while priming the immune system for a robust anti-tumor response. This dual functionality aligns with the emerging paradigm of integrating radiotherapy with immune checkpoint inhibitors for synergistic treatment outcomes.

The complexity of GBM requires a multi-faceted approach to therapy. Fractionated irradiation models have revealed that GBM cells adapt to radiation by acquiring stem-like properties, transitioning toward a mesenchymal phenotype, and enhancing DNA damage repair mechanisms—an adaptive process known as radioresistance. This process is driven by epigenetic modifications, alternative splicing, and posttranslational protein alterations. Given these challenges, the incorporation of adaptive radiotherapy (ART) with real-time functional imaging and nanotechnology could redefine treatment paradigms. By continuously monitoring tumor evolution and adjusting therapeutic strategies accordingly, ART, coupled with nanoparticle-based radiosensitization, holds the potential to overcome resistance mechanisms dynamically.

The complexity of GBM requires a multi-faceted approach to therapy. Fractionated irradiation models have revealed that GBM cells adapt to radiation by acquiring stem-like properties, transitioning toward a mesenchymal phenotype, and enhancing DNA damage repair mechanisms—an adaptive process known as radioresistance. To counteract these resistance mechanisms, stimuli-responsive nanomedicine provides an opportunity to modulate tumor biology dynamically. Recent research has demonstrated that integrating gold nanoparticle-based radiosensitizers with targeted immunotherapy can enhance radiation-induced tumor control while modulating the tumor microenvironment (TME) for sustained anti-cancer immunity [1].

Nanomedicine provides an opportunity to mitigate intrinsic and acquired resistance to RT in GBM. One of the most significant barriers to effective RT is hypoxia, which reduces radiation-induced reactive oxygen species (ROS) generation, diminishing DNA damage and limiting therapeutic efficacy. Stimuli-responsive nanocarriers, including hypoxia-activated prodrugs or radiosensitizers, can selectively release therapeutic agents in oxygen-deprived tumor regions, thereby restoring radiosensitivity. Gold nanoparticles (AuNPs) and other high-Z materials offer additional benefits by enhancing radiation dose deposition in tumor tissues while minimizing damage to surrounding healthy structures.

Another critical avenue involves targeting GBM’s redox imbalance. Elevated intracellular GSH levels neutralize ROS, contributing to radioresistance. Redox-responsive nanoparticles, functionalized with disulfide bonds, can selectively release their therapeutic payload in response to high GSH concentrations, amplifying oxidative stress in tumor cells and sensitizing them to radiation-induced DNA damage. These approaches exemplify how stimuli-responsive nanomedicine can transform GBM’s pathological microenvironment into a therapeutic target.

Recent advancements in oxygen-independent radiodynamic therapy (OIRDT) have introduced innovative approaches for overcoming tumor hypoxia limitations in RT. Unlike conventional radiation therapy, which relies heavily on oxygen availability to generate cytotoxic ROS, OIRDT leverages nanoparticles capable of inducing therapeutic effects independent of oxygen levels. Specifically, nanoparticles based on hafnium oxide, titanium dioxide, and rare earth oxides have demonstrated radiosensitizing capabilities through X-ray activation mechanisms [2]. Furthermore, nanoformulations such as albumin-based drug carriers and polymeric micelles have shown significant promise in improving the stability, bioavailability, and targeting efficacy of anti-GBM therapeutics [1,2,3]. These formulations enhance drug solubility and sustain release kinetics, optimizing therapeutic efficacy while minimizing systemic toxicity [3].

### 1.2. Integrating Nanomedicine with Emerging Radiotherapy Modalities

The integration of nanomedicine with advanced RT techniques, such as proton therapy, represents another frontier in GBM treatment. Proton therapy delivers highly localized radiation with minimal off-target effects, which aligns well with the precision-targeting capabilities of stimuli-responsive nanocarriers. The combination of these modalities could allow for radiation dose escalation within the tumor while sparing adjacent healthy tissue, ultimately improving the therapeutic index. Furthermore, theranostic platforms that combine diagnostic imaging with therapeutic delivery offer real-time monitoring of nanoparticle biodistribution and treatment efficacy, enabling truly adaptive therapy tailored to individual patients.

Given the vast inter- and intratumoral heterogeneity observed in GBM, the future of nanomedicine lies in patient-specific therapeutic strategies. Advances in multi-omics technologies and artificial intelligence (AI) have paved the way for personalized nanocarriers tailored to individual tumor profiles. For instance, nanoparticles functionalized with ligands targeting EGFRvIII, a GBM-specific mutation, could achieve unparalleled specificity in drug delivery. Additionally, integrating nanomedicine with liquid biopsy technologies—such as circulating tumor DNA (ctDNA) and exosomal analysis—could provide non-invasive, real-time tracking of treatment response and disease progression.

### 1.3. Novel Hypothesis: Leveraging Immune Modulation for Radiosensitization

We hypothesize that combining nanomedicine with immune-modulating strategies could revolutionize GBM treatment. Nanoparticles designed to release immune checkpoint inhibitors or cytokines in response to radiation-induced inflammation may convert the traditionally immunosuppressive TME into an immunostimulatory one. This approach aligns with emerging evidence that RT induces immunogenic cell death (ICD), bridging local tumor control with systemic immune activation.

Integrating stimuli-responsive nanomedicine with RT represents a paradigm shift in GBM treatment by targeting key challenges such as hypoxia, redox imbalances, and immune suppression. By leveraging the pathological hallmarks of GBM as therapeutic targets, these innovative platforms have the potential to significantly improve treatment efficacy, paving the way for truly personalized and adaptive oncological care. The following sections of this review will further explore the mechanisms, applications, and future directions of these transformative technologies.

**Figure 1:**
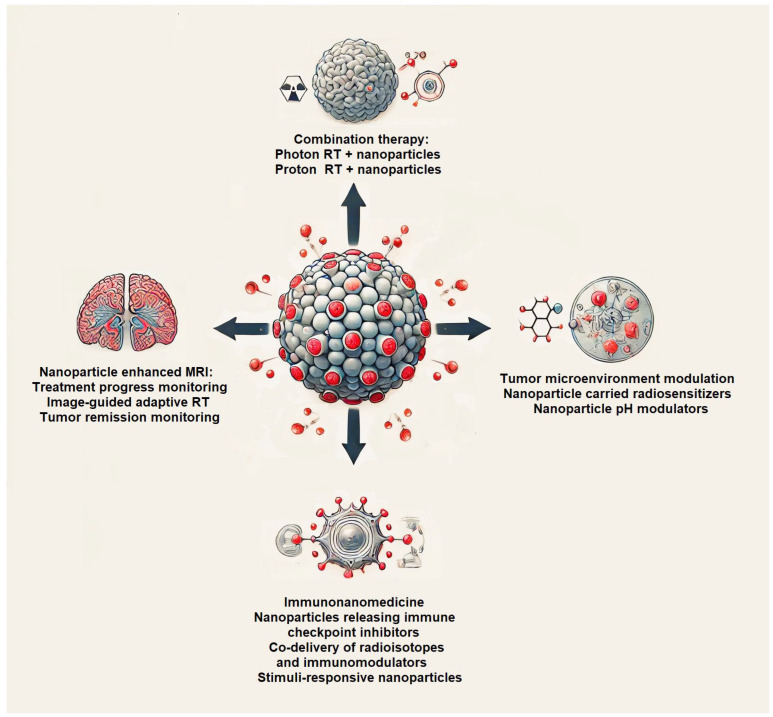
Nanomedicine-enhanced strategies for glioblastoma therapy. Schematic representation of core applications of nanomedicine in the treatment of glioblastoma.

Figure 1 presents a conceptual overview of nanomedicine-enhanced strategies for glioblastoma therapy. At the center is a glioblastoma tumor with four distinct applications of nanoparticles. These include radiosensitization through combination with photon or proton radiotherapy, modulation of the tumor microenvironment, immunonanomedicine approaches, nanoparticle-assisted MRI monitoring, and image-guided adaptive therapy. Figure 1 emphasizes the versatility of nanotechnology in targeting, treating, and monitoring glioblastoma with precision.

**Figure 2:**
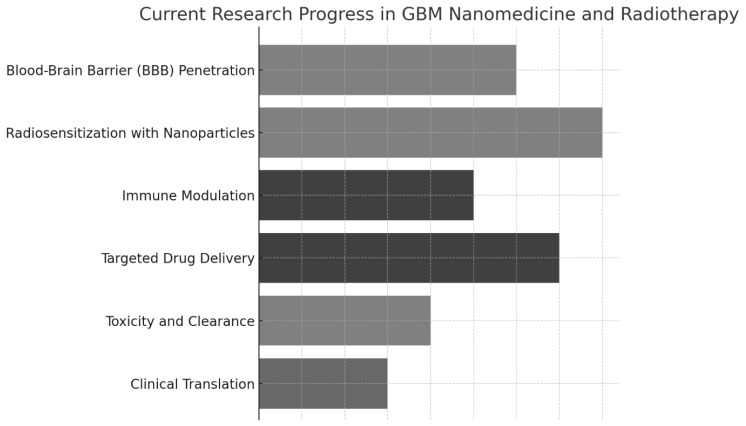
Advances in research in nanomedicine-enhanced radiotherapy for glioblastoma, including mechanisms of radiosensitization, tumor microenvironment targeting, and integration with adaptive radiotherapy modalities.

Figure 2 illustrates the current research progress in various areas of GBM nanomedicine and RT advancement (scores around 7–8), while clinical translation and toxicity/clearance remain relatively underdeveloped (scores around 3–4). These findings highlight the significant strides in improving radiation efficacy and drug targeting. They also emphasize the need for further research to translate these innovations into clinical practice and ensure their long-term safety.

## 2. Tumor Microenvironment as a Target for Stimuli-Responsive Nanomedicines

The GBM TME plays a decisive role in therapeutic resistance, making it an attractive target for precision nanomedicine. Hypoxia is one of the most well-documented factors that drive radioresistance in GBM, primarily by impairing the generation of radiation-induced ROS, which mediate DNA damage [4,5]. Hypoxia-activated nanoparticles designed to deliver radiosensitizers, such as nitroimidazole derivatives, selectively release their payload in oxygen-deprived regions, restoring radiation sensitivity and improving therapeutic outcomes [6]. These approaches have demonstrated promising results in preclinical models, particularly through the use of polymeric micelles and mesoporous silica nanoparticles incorporating hypoxia-cleavable bonds [7].

Beyond hypoxia, GBM’s acidic TME—driven by aberrant glycolysis and lactate accumulation—further complicates treatment response. Acid-responsive nanocarriers functionalized with zwitterionic polymers or hydrazone bonds have been developed to facilitate targeted drug release under acidic conditions, thereby increasing therapeutic efficacy while reducing systemic toxicity [8,9]. For example, polymeric nanogels capable of pH-sensitive swelling behavior have shown enhanced tumor penetration and controlled drug release, offering a promising avenue for combination therapies with RT [10].

Redox imbalances within GBM also present an opportunity for targeted interventions. Tumor cells exhibit high intracellular GSH levels, which neutralize ROS and contribute to radioresistance. Redox-sensitive nanoparticles engineered with disulfide bonds can undergo selective breakdown in the presence of high GSH concentrations, leading to precise drug release and enhanced oxidative stress within tumor cells [11]. Selenium-based nanoparticles and polymeric micelles with redox-sensitive crosslinks have demonstrated efficacy in preclinical GBM models, significantly improving radiosensitivity [12,13].

Nanomedicine also offers a powerful means to enhance RT through the use of high-Z materials such as gold nanoparticles (AuNPs), which amplify radiation dose deposition. Unlike conventional spherical AuNPs, engineered anisotropic nanostructures, such as gold nanopeanuts, have demonstrated superior radiosensitization effects by improving tumor penetration and enhancing secondary electron production during irradiation [14,15]. The integration of AuNPs into proton therapy has further improved the precision and efficacy of radiation dose deposition while minimizing collateral damage to healthy brain tissue [16].

Theranostic systems represent another exciting frontier in GBM management. Dual-functional nanocarriers combining imaging and therapeutic capabilities enable real-time monitoring of drug delivery and tumor response. Radiolabeled AuNPs have been integrated into multimodal imaging systems, offering simultaneous radiosensitization and imaging via computed tomography (CT) and magnetic resonance imaging (MRI) [17]. Upconversion nanoparticles capable of X-ray-activated drug release further highlight the potential of theranostic nanomedicine in GBM therapy [18].

Emerging research has highlighted the crucial role of the TGF-β signaling pathway in regulating glioma prognosis and therapeutic responses. Recent studies have identified a TGF-β-related long non-coding RNA (lncRNA) signature that can predict immune microenvironment interactions and glioma progression [19]. Understanding this signaling cascade has implications for integrating nanomedicine-enhanced radiotherapy with immunomodulatory strategies, particularly in leveraging checkpoint inhibitors and tumor-targeted nanocarriers [19].

Another promising avenue involves immune modulation, where certain stimuli-responsive nanoparticles have been developed to incorporate immune-activating agents. These systems synergize with RT-induced immunogenic cell death (ICD) by releasing checkpoint inhibitors or STING agonists in hypoxic or acidic regions of the TME [20]. For example, polymeric nanoparticles co-delivering PD-1 inhibitors and Toll-like receptor (TLR) agonists have shown remarkable efficacy in enhancing anti-tumor immune responses in preclinical studies [21].

The blood–brain barrier (BBB) remains one of the most formidable obstacles in the treatment of GBM, acting as a tightly regulated gatekeeper that severely limits the penetration of therapeutics into the brain parenchyma. While the BBB is often compromised in high-grade gliomas, its permeability is highly heterogeneous, with intact regions impeding drug transport and reducing the efficacy of systemic therapies [5]. Conventional small-molecule chemotherapies, while capable of limited BBB penetration, often fail to achieve therapeutic concentrations within the tumor core and infiltrative margins, necessitating the development of novel strategies to enhance drug delivery.

Recent advancements in receptor-mediated transcytosis (RMT) have enabled nanoparticles to leverage endogenous transport mechanisms to traverse the BBB. Functionalizing nanoparticles with ligands targeting transferrin receptors (TfR), insulin receptors (IR), or low-density lipoprotein receptors (LDLR) has demonstrated increased BBB penetration in preclinical GBM models [6]. Another emerging approach involves the use of focused ultrasound (FUS) with microbubble cavitation, which temporarily disrupts the BBB, allowing for enhanced nanoparticle permeability and retention within GBM tissues [7]. However, concerns regarding the safety and reproducibility of FUS-mediated BBB modulation remain, necessitating further clinical validation [8].

Additionally, peptide-functionalized nanoparticles incorporating BBB-penetrating moieties, such as Angiopep-2 or TAT peptides, have shown promise in improving drug transport across endothelial cells [9]. These engineered nanocarriers capitalize on receptor-ligand interactions to facilitate transcytosis while minimizing systemic toxicity. Future iterations of BBB-targeting nanomedicines may integrate multi-ligand functionalization, combining different targeting elements to optimize specificity and efficiency [10].

Beyond BBB penetration, safety, toxicity, and long-term biocompatibility represent critical challenges in the clinical translation of nanomedicines for GBM. Unlike small-molecule drugs, nanoparticles exhibit prolonged circulation times and enhanced cellular uptake, which can lead to off-target accumulation and systemic toxicity [11]. In particular, metal-based nanoparticles, such as gold and silver nanostructures, have been associated with oxidative stress, complement system activation, and inflammatory responses in vivo [12]. These concerns have driven a shift toward biodegradable and bio-inspired nanocarriers, including lipid-based, polymeric, and exosome-derived formulations, which offer improved clearance profiles and reduced long-term toxicity [13].

To mitigate the risks associated with nanoparticle accumulation, researchers are actively exploring “self-destructing” nanocarriers that undergo controlled degradation after achieving their therapeutic effect. These systems utilize stimuli-responsive materials that degrade in response to physiological cues, such as pH shifts, enzymatic activity, or oxidative stress, ensuring minimal residual toxicity [14]. Furthermore, the integration of exosome-based nanomedicines, which mimic endogenous vesicle transport mechanisms, presents a promising avenue for enhancing biocompatibility and immune evasion [15].

Future directions in GBM nanomedicine should prioritize the development of dual-targeting strategies, integrating BBB-penetrating elements with tumor-specific recognition moieties to enhance selectivity and efficacy. Additionally, leveraging artificial intelligence (AI) and computational modeling could accelerate the optimization of nanoparticle design, predicting biodistribution, clearance, and therapeutic outcomes with greater precision [16]. Standardizing preclinical-to-clinical pipelines, including advanced imaging techniques for nanoparticle tracking in vivo and the use of patient-derived xenograft (PDX) models, will be essential for translating promising nanomedicine strategies into viable GBM treatments [17].

## 3. Blood–Brain Barrier (BBB) Penetration and Drug Delivery

The blood–brain barrier (BBB) remains one of the most formidable obstacles in the treatment of GBM, acting as a tightly regulated gatekeeper that severely limits the penetration of therapeutics into the brain parenchyma. While the BBB is often compromised in high-grade gliomas, its permeability is highly heterogeneous, with intact regions impeding drug transport and reducing the efficacy of systemic therapies [17]. Conventional small-molecule chemotherapies, while capable of limited BBB penetration, often fail to achieve therapeutic concentrations within the tumor core and infiltrative margins, necessitating the development of novel strategies to enhance drug delivery.

Recent advancements in receptor-mediated transcytosis (RMT) have enabled nanoparticles to leverage endogenous transport mechanisms to traverse the BBB. Functionalizing nanoparticles with ligands targeting transferrin receptors (TfR), insulin receptors (IR), or low-density lipoprotein receptors (LDLR) has demonstrated increased BBB penetration in preclinical GBM models [18]. Another emerging approach involves the use of focused ultrasound (FUS) with microbubble cavitation, which temporarily disrupts the BBB, allowing for enhanced nanoparticle permeability and retention within GBM tissues [19]. However, concerns regarding the safety and reproducibility of FUS-mediated BBB modulation remain, necessitating further clinical validation [20].

Additionally, peptide-functionalized nanoparticles incorporating BBB-penetrating moieties, such as Angiopep-2 or TAT peptides, have shown promise in improving drug transport across endothelial cells [20]. These engineered nanocarriers capitalize on receptor-ligand interactions to facilitate transcytosis while minimizing systemic toxicity. Future iterations of BBB-targeting nanomedicines may integrate multi-ligand functionalization, combining different targeting elements to optimize specificity and efficiency [21].

Beyond BBB penetration, safety, toxicity, and long-term biocompatibility represent critical challenges in the clinical translation of nanomedicines for GBM. Unlike small-molecule drugs, nanoparticles exhibit prolonged circulation times and enhanced cellular uptake, which can lead to off-target accumulation and systemic toxicity [22]. In particular, metal-based nanoparticles, such as gold and silver nanostructures, have been associated with oxidative stress, complement system activation, and inflammatory responses in vivo [22,23]. These concerns have driven a shift toward biodegradable and bio-inspired nanocarriers, including lipid-based, polymeric, and exosome-derived formulations, which offer improved clearance profiles and reduced long-term toxicity [23].

To mitigate the risks associated with nanoparticle accumulation, researchers are actively exploring “self-destructing” nanocarriers that undergo controlled degradation after achieving their therapeutic effect. These systems utilize stimuli-responsive materials that degrade in response to physiological cues, such as pH shifts, enzymatic activity, or oxidative stress, ensuring minimal residual toxicity [24]. Furthermore, the integration of exosome-based nanomedicines, which mimic endogenous vesicle transport mechanisms, presents a promising avenue for enhancing biocompatibility and immune evasion [25].

## 4. Exploiting Extracellular Vesicles (EVs) for Nanomedicine Delivery

Extracellular vesicles (EVs) have emerged as one of the most promising nanocarriers for GBM therapy, offering unique advantages in overcoming the challenges associated with blood–brain barrier (BBB) penetration and tumor-specific targeting. EVs, including exosomes and microvesicles, exhibit intrinsic biocompatibility, low immunogenicity, and a natural ability to transport bioactive molecules such as proteins, nucleic acids, and lipids. These properties make them ideal candidates for the delivery of therapeutics across the BBB, an obstacle that has hindered conventional nanoparticle-based treatments [26,27].

Hybrid EV-nanoparticle systems have garnered increasing interest due to their ability to combine the stability and high drug-loading capacity of synthetic nanocarriers with the biocompatibility and targeting efficiency of extracellular vesicles. Recent studies demonstrate that EV-coated nanoparticles exhibit superior blood–brain barrier (BBB) penetration and sustained tumor retention, making them promising vehicles for glioblastoma-targeted therapy [27].

One of the critical advantages of EV-based drug delivery is their ability to be engineered with tumor-targeting ligands, thereby improving their specificity for GBM cells while minimizing off-target effects. Studies have demonstrated that EVs functionalized with integrin-targeting peptides or transferrin receptor ligands significantly enhance their uptake by GBM cells, facilitating the intracellular delivery of chemotherapeutic agents and radiosensitizers [28]. Additionally, genetic modifications that incorporate surface-displayed ligands such as folate or epidermal growth factor receptor (EGFR)-targeting peptides have shown promising results in preclinical models by improving the selectivity and accumulation of EVs within the tumor microenvironment [29].

Hybrid EV-nanoparticle systems have also garnered increasing interest as they combine the stability and high drug-loading capacity of synthetic nanocarriers with the biological advantages of EVs. By coating traditional lipid or polymeric nanoparticles with EV membranes, researchers have successfully enhanced drug bioavailability and tumor specificity in GBM treatment [28]. This biomimetic approach not only prolongs the circulation half-life of therapeutic agents but also enables them to evade immune surveillance, thus improving their therapeutic index [29]. Furthermore, incorporating stimuli-responsive elements into hybrid EV-nanoparticles allows for controlled drug release in response to tumor-specific cues such as acidic pH or hypoxia, further enhancing their efficacy [30].

A promising frontier in EV-based nanomedicine is the utilization of patient-derived EVs for personalized therapy. Given the heterogeneity of GBM, patient-specific EVs carrying individualized molecular cargo could revolutionize treatment by delivering tailored therapeutics that target unique tumor signatures. Recent research has demonstrated that patient-derived exosomes can be loaded with gene-editing tools such as CRISPR/Cas9 to selectively silence oncogenic pathways within GBM cells, providing a highly precise therapeutic approach [31]. Additionally, the use of autologous EVs mitigates the risk of immune rejection, making them an attractive option for future clinical applications [32].

Despite these advancements, several challenges remain in translating EV-based therapies to the clinic. One major hurdle is the large-scale production and standardization of EVs with consistent quality and therapeutic efficacy. Current isolation methods, such as ultracentrifugation and size-exclusion chromatography, lack scalability, necessitating the development of more efficient purification techniques for clinical-grade EV production [33]. Another limitation is the rapid clearance of EVs from circulation, which reduces their bioavailability and necessitates strategies to prolong their systemic half-life. Engineering EVs with polyethylene glycol (PEG) coatings or incorporating fusion proteins that enhance circulation time may address these pharmacokinetic challenges [34].

## 5. Combination Therapy

### 5.1. Harnessing Immuno-Nanomedicine for Combination Therapies

Advances in immuno-nanomedicine are revolutionizing the treatment of GBM by integrating nanotechnology with immunotherapeutic strategies to modulate the tumor microenvironment and enhance radiation therapy. Nanoparticles engineered to release immune checkpoint inhibitors, cytokines, or tumor vaccines in response to tumor-specific stimuli hold the potential to transform radiotherapy into an immunostimulatory modality. The immunosuppressive nature of the GBM microenvironment, driven by regulatory T cells (Tregs), tumor-associated macrophages (TAMs), and myeloid-derived suppressor cells (MDSCs), creates a major barrier to effective immune activation. Nanocarriers functionalized with PD-1/PD-L1 inhibitors can disrupt these pathways, reinvigorating the anti-tumor immune response while minimizing systemic toxicity [35].

A particularly promising approach involves the co-delivery of radioisotopes and immunomodulators within nanoparticle formulations. Alpha-emitting radioisotopes such as actinium-225 and astatine-211 have demonstrated significant potential in inducing localized tumor destruction while simultaneously triggering immunogenic cell death (ICD), which enhances dendritic cell activation and promotes long-term tumor immunity [36]. These strategies leverage the concept of in situ vaccination, wherein the tumor microenvironment itself is remodeled to become an antigen-rich site capable of eliciting systemic immune responses.

Stimuli-responsive nanoparticles designed for the spatiotemporal release of immunotherapies further expand the capabilities of nanomedicine in GBM. By utilizing hypoxia- or pH-sensitive carriers, the precise release of immune-stimulatory agents such as IL-12, GM-CSF, or STING agonists can be achieved within the TME, ensuring maximal immune activation while sparing healthy tissues [37]. Recent studies have demonstrated that polymeric nanoparticles loaded with Toll-like receptor (TLR) agonists effectively convert TAMs from an M2 immunosuppressive phenotype to an M1 pro-inflammatory phenotype, enhancing the efficacy of RT and reversing immune evasion mechanisms in GBM [38].

Nanoparticle-based cancer vaccines offer yet another innovative strategy, where synthetic carriers encapsulate tumor-associated antigens alongside immune adjuvants to prime cytotoxic T cell responses. Lipid-based nanovaccines delivering neoantigens in combination with RT have shown remarkable preclinical success, leading to enhanced tumor regression and prolonged survival in GBM models. Personalized nanovaccine approaches utilizing patient-derived tumor antigens are now being explored in clinical settings with the goal of generating individualized anti-tumor immunity [39].

While these immuno-nanomedicine approaches are highly promising, several translational challenges must be addressed. The heterogeneity of GBM poses difficulties in identifying universal antigenic targets, necessitating further research into patient-specific immune signatures. Additionally, the optimal combination of RT, nanomedicine, and immunotherapy must be determined to maximize synergy while minimizing immune-related toxicities [40]. Recent advancements in nanomedicine have led to the commercialization of several nanoparticle-based radiosensitizers. Notably, AGuIX (NH TherAguix) and NBTXR3 (Nanobiotix) are two promising nanoparticles currently in clinical trials for enhancing RT in glioblastoma patients. These agents leverage high atomic number elements to amplify radiation-induced DNA damage selectively within tumor tissues. Additionally, several patents have been filed concerning stimuli-responsive nanoparticles for cancer therapy, highlighting the growing interest in this field

### 5.2. Nanoparticle-Driven Reprogramming of Cancer Stem Cells (CSCs)

Cancer stem cells (CSCs) represent a highly aggressive and therapy-resistant subpopulation within GBM, contributing to tumor recurrence and treatment failure. These cells exhibit self-renewal properties and plasticity, allowing them to adapt to environmental stressors, including RT and chemotherapy. Conventional therapies fail to eradicate CSCs due to their enhanced DNA repair mechanisms, low proliferation rates, and their localization within protective tumor niches. Stimuli-responsive nanomedicine offers a promising strategy to overcome these barriers by enabling the targeted modulation of CSC biology [41].

One approach involves the development of nanocarriers functionalized with ligands that selectively bind to CSC surface markers, such as CD133, CD44, and L1CAM, to ensure precise drug delivery while sparing normal neural stem cells. These nanoparticles can be engineered to release epigenetic modulators, such as histone deacetylase inhibitors or DNA methyltransferase inhibitors, in response to tumor-specific stimuli like hypoxia or acidic pH. By disrupting CSC epigenetic plasticity, these nanotherapeutics can induce differentiation, rendering the cells more susceptible to standard treatments [42].

In addition, nanoparticles designed for the controlled release of differentiation-inducing agents, such as retinoic acid or bone morphogenetic proteins, have shown efficacy in promoting CSC differentiation, thereby reducing their tumorigenic potential. Combining these strategies with radiosensitizers or chemotherapeutics within multifunctional nanocarriers has demonstrated synergistic effects, effectively eradicating both CSCs and the bulk tumor cell population. Preclinical models have shown that CSC-targeted nanomedicine significantly reduces tumor regrowth and prolongs survival compared to conventional therapies alone [42,43].

### 5.3. Integrating Nanomedicine with Proton Therapy

Proton therapy has emerged as a promising modality for GBM treatment due to its superior dose distribution profile, characterized by the Bragg peak effect, which enables maximal energy deposition at a well-defined tumor depth while sparing surrounding healthy tissues. However, the therapeutic efficacy of proton therapy remains limited by the radioresistance of GBM cells and the hypoxic nature of the tumor microenvironment, which necessitates novel approaches to enhance its impact. The integration of nanomedicine, particularly gold nanoparticles (AuNPs), into proton therapy is a rapidly expanding research area aimed at overcoming these limitations [44].

Gold nanoparticles, due to their high atomic number and strong interaction with ionizing radiation, have been widely investigated as radiosensitizers. Their ability to enhance local energy deposition when irradiated with protons has been demonstrated in preclinical models. Recent advances have explored the influence of nanoparticle shape, size, and functionalization on radiosensitization. Anisotropic AuNPs, such as gold nanopeanuts, have exhibited superior radiosensitizing properties compared to their spherical counterparts due to their increased surface area and enhanced cellular uptake [45]. In a recent study, gold nanopeanuts conjugated with cisplatin were shown to significantly enhance DNA damage and ROS generation in GBM cells when combined with X-ray irradiation, underscoring their potential in nano-chemo-radiotherapy [11].

Furthermore, Monte Carlo simulations have been employed to optimize the size and concentration of AuNPs for proton therapy applications. Studies suggest that AuNPs within the 10–25 nm range provide the optimal balance between physical dose enhancement and biological interactions, maximizing DNA double-strand breaks while minimizing off-target toxicity. This size range ensures efficient tumor penetration and retention, further amplifying the therapeutic efficacy of proton therapy [46].

Beyond physical dose enhancement, the combination of AuNPs with proton therapy has been explored for its potential to modulate the GBM tumor microenvironment. Functionalized AuNPs can be engineered to carry radiosensitizing agents, hypoxia-activated prodrugs, or immune-stimulatory molecules, thereby transforming the traditionally radioresistant GBM into a more susceptible phenotype. For instance, recent investigations have demonstrated that AuNPs conjugated with redox-modulating agents can disrupt the tumor’s antioxidant defense mechanisms, sensitizing GBM cells to proton irradiation-induced oxidative stress [47].

Despite these promising findings, several challenges remain in translating AuNP-enhanced proton therapy into clinical practice. The biodistribution and clearance of nanoparticles must be carefully assessed to minimize systemic toxicity. Additionally, the potential interactions between AuNPs and different proton energy levels need further exploration to optimize treatment parameters. Future research should focus on developing multifunctional AuNP platforms that integrate real-time imaging capabilities for theranostic applications, enabling precision-guided proton therapy.

### 5.4. Integrating Nanomedicine with X-Ray Radiotherapy

X-ray radiotherapy remains the most widely used modality for GBM treatment, but its efficacy is often compromised by tumor hypoxia, radioresistance, and collateral damage to healthy brain tissue. The integration of nanomedicine into X-ray radiotherapy has been investigated to enhance therapeutic efficacy by exploiting the unique interactions between high-Z nanoparticles and ionizing radiation. Unlike proton therapy, where energy deposition is highly localized through the Bragg peak, X-ray photons primarily interact with matter via the Compton and photoelectric effects, leading to dose enhancement through secondary electron production and Auger cascades [48].

Gold nanoparticles (AuNPs), due to their high atomic number, efficiently absorb X-rays and generate a localized dose amplification effect. When irradiated with kilovoltage or megavoltage X-rays, AuNPs increase the production of secondary electrons, including low-energy electrons that contribute to DNA double-strand breaks and oxidative stress in tumor cells. This phenomenon enhances tumor cytotoxicity while sparing adjacent normal tissues. The effectiveness of AuNPs in radiosensitization depends on their size, concentration, and intracellular localization. Studies have shown that AuNPs within the 1–5 nm range exhibit optimal uptake and DNA proximity, thereby maximizing their radiosensitizing effects [49].

Recent advancements in nanomedicine have explored functionalized AuNPs conjugated with radiosensitizers, such as cisplatin or PARP inhibitors, to further amplify DNA damage. Additionally, bimetallic nanoparticles, including gold-silver and hafnium-based systems, have demonstrated superior radio-enhancing properties compared to monometallic formulations. Preclinical studies have confirmed that these nanoparticles significantly enhance tumor control when combined with fractionated X-ray radiotherapy [50].

Beyond dose enhancement, the interaction of nanoparticles with X-ray radiation has been exploited to generate ROS, further intensifying tumor cell damage. Functionalized nanocarriers delivering ROS-inducing agents, such as titanium dioxide and cerium oxide nanoparticles, have demonstrated increased radiation-induced oxidative stress and apoptosis in GBM models. These strategies are particularly relevant for overcoming the radioresistance associated with hypoxic tumor regions [12].

Research is conducted on optimizing nanoparticle composition, surface chemistry, and administration routes to improve tumor specificity and minimize systemic toxicity. The use of advanced Monte Carlo simulations to refine nanoparticle-based radiotherapy protocols could enhance the precision and safety of X-ray-enhanced nanomedicine for GBM treatment [51]. Clinical translation will require further validation in large-scale trials to ensure the efficacy and safety of nanoparticle-assisted radiotherapy in GBM patients.

## 6. Nanomedicine in Clinical Trials

Several clinical trials are evaluating the efficacy of nanomedicine-enhanced radiotherapy in GBM treatment. As of 2025, multiple ongoing studies are focusing on improving the therapeutic index of radiation therapy using nanomedicine approaches.

For instance, NCT04899989, expected to conclude in late 2026, is assessing the combination of gold nanoparticle-based radiosensitizers with standard RT. This phase II trial aims to determine whether gold nanoparticles enhance radiation-induced DNA damage selectively in glioblastoma cells while reducing off-target toxicity in normal brain tissue. Similarly, NCT04295294, set to conclude in early 2027, investigates lipid-based nanocarriers designed to penetrate the blood–brain barrier (BBB) efficiently. This study explores whether these nanocarriers can improve the bioavailability of temozolomide, the current standard chemotherapeutic agent, thereby enhancing radiosensitization.

Another key clinical study, NCT05075439, is evaluating the combination of hypoxia-responsive nanoparticles with fractionated proton therapy, with an anticipated completion date in 2028. This trial is designed to address tumor hypoxia, a major barrier to effective radiotherapy, by selectively releasing radiosensitizing agents within low-oxygen tumor regions. Preliminary findings suggest that this approach significantly enhances ROS generation, improving tumor control rates.

Beyond radiosensitization, some trials focus on immune modulation. NCT05123352, concluding in 2026, is investigating the combination of checkpoint inhibitors and nanomedicine-enhanced RT. This study hypothesizes that nanoparticles engineered to co-deliver PD-L1 inhibitors alongside radiation therapy will amplify the immune response, thereby improving long-term tumor suppression.

Additionally, theranostic platforms integrating real-time imaging with nanoparticle-enhanced RT are being tested in NCT05320890. This study, ending in 2027, explores whether superparamagnetic iron oxide nanoparticles (SPIONs) can serve as both MRI contrast agents and radiosensitizers, enabling precise tumor tracking and personalized dose adaptation.

These clinical trials collectively provide crucial insights into the translational potential of nanotechnology-driven approaches in radiation oncology. While preliminary results are promising, challenges remain in ensuring long-term safety, optimizing dosing strategies, and validating clinical efficacy across diverse patient populations. Continued advancements in nanoparticle design, AI-driven optimization, and multimodal therapeutic integration will be essential for the successful implementation of nanomedicine-enhanced RT in clinical practice.” “Several clinical trials are evaluating the efficacy of nanomedicine-enhanced radiotherapy in GBM treatment. For example, NCT04899989 is assessing the combination of gold nanoparticle-based radiosensitizers with standard RT, while NCT04295294 is investigating lipid-based nanocarriers for BBB penetration in glioblastoma patients. These studies provide crucial insights into the translational potential of nanotechnology-driven approaches in radiation oncology.

Table 1 summarizes an overview of advancements in glioblastoma nanomedicine, key preclinical studies, ongoing clinical trials, and marketed nanoparticle-based products. This compilation highlights the translational progression of nanotechnology-driven therapies from experimental models to clinical applications, emphasizing their potential to improve patient outcomes.

## 7. Nanomedicine for Non-Invasive Monitoring of Treatment Progression

The integration of nanomedicine with radiation therapy (RT), including both proton therapy and X-ray radiotherapy, extends beyond treatment enhancement to non-invasive monitoring of therapeutic response. As adaptive RT continues to evolve, the use of nanoparticle-based diagnostic agents enables real-time assessment of tumor progression and therapeutic efficacy, facilitating personalized treatment adjustments. Theranostic nanoparticles, which combine both imaging and therapeutic functions, are particularly promising in this regard [53].

MRI-visible nanocarriers have been developed to track nanoparticle biodistribution within GBM tissues, offering a means to assess drug penetration and radiosensitization effects. Superparamagnetic iron oxide nanoparticles (SPIONs) conjugated with radiosensitizers have demonstrated strong MRI contrast enhancement while simultaneously amplifying radiation-induced DNA damage in tumor cells. These platforms allow clinicians to evaluate nanoparticle accumulation in GBM regions and correlate localization with treatment efficacy [53,54].

Beyond MRI, multimodal imaging nanoparticles incorporating positron emission tomography (PET), computed tomography (CT), and fluorescence capabilities offer a comprehensive approach to tracking tumor response. Gold nanoparticles functionalized with radiotracers have been explored for PET-guided adaptive RT, enabling real-time adjustments in radiation dosing based on tumor metabolic activity. Hybrid nanoparticles, such as gadolinium-doped gold nanoclusters, have also shown promise in integrating MRI and CT contrast enhancement, facilitating simultaneous radiosensitization and precise tumor delineation during RT planning [55].

A key advancement in nanotheranostics is the utilization of nanoparticles for liquid biopsy applications. Circulating tumor DNA (ctDNA) and extracellular vesicle-based biomarkers offer a minimally invasive method for monitoring tumor evolution throughout RT treatment. Nanoparticle-enabled ctDNA capture platforms enhance the sensitivity of detecting tumor-derived genetic alterations, providing insights into treatment resistance mechanisms. These approaches may allow for early identification of tumor adaptation to radiation, guiding the implementation of dose adjustments or combination therapies [56].

Additionally, adaptive RT strategies leveraging nanoparticle-based tumor characterization are under investigation. Functionalized nanoparticles capable of detecting intratumoral hypoxia, a key driver of radioresistance, can be employed to dynamically adjust radiation dose fractionation. Oxygen-releasing nanoparticles have been proposed as dual-purpose agents that mitigate hypoxia while enhancing radiosensitivity, ultimately improving RT outcomes in GBM patients. Such innovations enable the transition from static RT regimens to highly individualized, real-time treatment adaptations based on nanoparticle-driven tumor profiling [57].

## 8. AI-Driven Nanomedicine in GBM Therapy

AI is transforming nanomedicine by accelerating the discovery of novel nanoparticle formulations and optimizing their interactions with the tumor TME. The AI-driven models analyze vast datasets to predict the most effective nanoparticle compositions for tumor targeting, immune modulation, and radiosensitization. By simulating in vivo interactions, AI aids in designing nanoparticles that selectively accumulate within hypoxic tumor regions, trigger immune activation, or alter metabolic pathways to enhance therapeutic outcomes [58].

AI also plays a pivotal role in understanding nanomedicine-induced immune responses by integrating multi-omics data to predict patient-specific immunogenicity. Machine learning algorithms assess how nanoparticles influence cytokine release, antigen presentation, and immune cell infiltration within the TME. These insights enable the development of immuno-nanomedicine approaches that synergize with radiation therapy to elicit durable anti-tumor responses [59].

In adaptive RT, AI enhances precision by integrating nanoparticle-enhanced imaging data with real-time tumor response assessments. AI-driven algorithms refine dose adaptation strategies by analyzing nanoparticle biodistribution, hypoxia status, and radiosensitization effects. This dynamic approach ensures that RT is continuously optimized, maximizing therapeutic efficacy while minimizing damage to healthy tissues [60,61].

The regulatory approval remains a major barrier to AI in clinical adoption. Current experimental research is focused on verifying AI-driven nanoparticle propositions by subjecting AI-designed nanocarriers to rigorous immunointeraction testing. These nanoparticles, optimized computationally for tumor-specific binding and immune evasion, are first validated in vitro to assess their targeting efficiency, biocompatibility, and therapeutic efficacy. Successful candidates then should undergo in vivo studies to evaluate biodistribution, clearance, and tumor response under physiological conditions. This stepwise approach ensures that AI-derived nanoparticle designs align with biological realities, refining their translation from theoretical models to clinically relevant therapeutics. Establishing standardized protocols for AI-driven nanoparticle validation will be essential in accelerating their clinical integration and regulatory approval. AI models are excellent at processing vast datasets, identifying patterns, and predicting outcomes, but they still lack the nuanced understanding, ethical judgment, and contextual adaptability that human scientists and clinicians provide [52,62].

Our concept of a “human-AI verificator” would be a compelling framework for the future of medical research and practice. In this proposal, AI would act as a high-efficiency analytical tool, generating hypotheses, predicting nanoparticle interactions, and optimizing treatment protocols. However, human scientists and experts would be the arbiters of validation—designing experiments, verifying AI-generated findings in the biological systems, and refining the models based on experimental feedback.

This approach could drastically accelerate research in nanomedicine-enhanced therapies, particularly in areas like glioblastoma treatment, where AI-driven nanoparticle design could be rapidly tested through in vitro and in vivo models. AI might propose nanocarriers with predicted immune evasion properties, but only rigorous laboratory testing can confirm their safety and therapeutic efficiency. As scientists, our role is to scrutinize AI-proposed designs and ensure that computational advancements translate into meaningful clinical breakthroughs.

## 9. Conclusions

Nanomedicine is redefining the way we approach GBM treatment, transforming radiation therapy into a more precise, personalized, and adaptable intervention. By leveraging stimuli-responsive nanocarriers, we can exploit the unique vulnerabilities of the tumor microenvironment—hypoxia, acidity, and redox imbalances—to maximize the impact of radiotherapy while reducing systemic toxicity. These advancements mark a significant step toward safer, more effective treatments that respond dynamically to the evolving nature of the disease.

The integration of proton and X-ray radiotherapy with nanotechnology is unlocking new possibilities for improving dose distribution, enhancing tumor targeting, and modulating the TME for better therapeutic outcomes. Gold-based nanostructures, particularly anisotropic nanoparticles such as gold nanopeanuts, are demonstrating superior radiosensitization properties, amplifying radiation-induced DNA damage while limiting harm to healthy tissues. AI-driven modeling is further pushing the boundaries of nanomedicine, optimizing nanoparticle design and refining their interactions with GBM cells and the immune system to elicit stronger anti-tumor responses.

Ongoing clinical trials are validating the effectiveness of gold nanoparticles, lipid-based carriers, and theranostic platforms, offering promising prospects for overcoming glioblastoma treatment resistance. Despite these advancements, challenges persist in ensuring long-term biocompatibility, scalable production, and clinical standardization. Future research must refine nanocarrier safety, enhance multimodal imaging integration, and establish regulatory frameworks to accelerate clinical translation.

Despite these promising advances, significant challenges remain. The complexity of the GBM microenvironment and the variability in patient responses make clinical translation an ongoing hurdle. Researchers should explore nanocarrier formulations for improved safety and biodistribution while integrating multi-omics technologies and AI-driven analytics to create truly personalized therapies. As these innovations continue to evolve, nanomedicine and radiation therapy together offer renewed hope for improving patient survival and shifting the treatment paradigm for GBM.

## Figures and Tables

**Table 1:** Summary of relevant studies, clinical trials, and marketed nanoparticle-based Products in glioblastoma therapy.

Category	Study/Trial/Product	Key Findings/Description	Reference/Status
Preclinical Studies	Hafnium Oxide Nanoparticles	Demonstrated enhanced radiosensitization effects through X-ray activation in glioblastoma models.	Babu et al., 2023 [14]
	Titanium Dioxide-Based Nanocarriers	Shown to improve drug delivery and radiation response in hypoxic glioblastoma environments.	Liu et al., 2017 [5]
	Hybrid EV-Nanoparticle Systems	Increased blood–brain barrier penetration and sustained tumor retention.	Du et al., 2023 [52]
Clinical Trials	NCT04899989	Evaluating gold nanoparticle-based radiosensitizers in combination with standard radiotherapy. Expected completion: 2026.	Ongoing
	NCT04295294	Investigating lipid-based nanocarriers to enhance temozolomide delivery and improve radiosensitization. Expected completion: 2027.	Ongoing
	NCT05075439	Testing hypoxia-responsive nanoparticles with fractionated proton therapy to enhance reactive oxygen species generation. Expected completion: 2028.	Ongoing
	NCT05123352	Studying co-delivery of PD-L1 inhibitors via nanoparticle platforms to amplify immune responses. Expected completion: 2026.	Ongoing
	NCT05320890	Exploring superparamagnetic iron oxide nanoparticles (SPIONs) for tumor imaging and radiosensitization. Expected completion: 2027.	Ongoing
Marketed Nanoparticle-Based Products	AGuIX (NH TherAguix)	Nanoparticle-based radiosensitizer enhancing radiation-induced DNA damage selectively within tumors.	In Clinical Trials
	NBTXR3 (Nanobiotix)	High-Z element nanoparticle system improving radiation therapy precision in glioblastoma treatment.	In Clinical Trials
	RNL-001 (Plus Therapeutics)	Nanoliposomal formulation of radioactive rhenium-186 for localized radiotherapy.	Ongoing Clinical Studies
